# An interpretable machine learning model using multimodal pretreatment features predicts pathological complete response to neoadjuvant immunochemotherapy in esophageal squamous cell carcinoma

**DOI:** 10.3389/fimmu.2025.1660897

**Published:** 2025-09-16

**Authors:** Xueping Wang, Wencheng Tan, Hui Sheng, Wenjia Zhou, Hailin Zheng, Kewei Huang, Jinfei Lin, Songhe Guo, Minjie Mao

**Affiliations:** ^1^ Department of Laboratory Medicine, Sun Yat-Sen University Cancer Center, Guangzhou, China; ^2^ State Key Laboratory of Oncology in South China, Guangdong Provincial Clinical Research Center for Cancer, Sun Yat-sen University Cancer Center, Guangzhou, China; ^3^ Department of Endoscopy, Sun Yat-Sen University Cancer Center, Guangzhou, China; ^4^ Department of Experimental Research, Sun Yat-Sen University Cancer Center, Guangzhou, China; ^5^ School of Medical Technology, Guangdong Medical University, Dongguan, China; ^6^ Guangzhou Institute of Cancer Research, the Affiliated Cancer Hospital, Guangzhou Medical University, Guangzhou, China

**Keywords:** esophageal squamous cell carcinoma, neoadjuvant immunochemotherapy, pathological complete response, machine learning, model interpretability

## Abstract

**Background:**

Although neoadjuvant immunochemotherapy (nICT) has revolutionized the management of locally advanced esophageal squamous cell carcinoma (ESCC), the inability to accurately predict pathological complete response (pCR) remains a major barrier to treatment personalization. We aimed to develop and validate an interpretable machine learning (ML) model using pretreatment multimodal features to predict pCR prior to nICT initiation.

**Methods:**

In this retrospective study, 114 ESCC patients receiving nICT were randomly allocated into training (n=81) and validation (n=33) cohorts (7:3 ratio). Predictors of pCR were identified from pretreatment clinical variables, endoscopic ultrasonography, and hematological biomarkers via least absolute shrinkage and selection operator (LASSO) regression. Eight machine learning algorithms were implemented to construct prediction models. Model performance was assessed by area under the receiver operating characteristic curve (AUC), sensitivity, specificity, positive predictive value (PPV), and negative predictive value (NPV). Shapley Additive Explanations (SHAP) provided feature importance and model interpretability.

**Results:**

Following feature selection, 17 variables were incorporated into model construction. The Random Forest (RF) model demonstrated perfect discrimination in the training cohort (AUC = 1.000, sensitivity = 1.000, specificity = 1.000, PPV = 1.000, NPV = 1.000), while maintaining robust predictive ability in the independent validation cohort (AUC = 0.913, sensitivity = 0.733, specificity = 0.889, PPV = 0.846, NPV = 0.800). Decision curve analysis (DCA) confirmed favorable clinical utility. SHAP analysis identified alcohol consumption, circumferential involvement ≥50%, elevated neutrophil-to-lymphocyte ratio (NLR), C-reactive protein (CRP), and alanine aminotransferase (ALT) as the key contributors to pCR prediction.

**Conclusions:**

We established a clinically applicable, interpretable ML model that accurately predicts pCR to nICT in ESCC by integrating multimodal pretreatment data. This tool may optimize patient selection for nICT and advance precision therapy paradigms.

## Introduction

Esophageal squamous cell carcinomas (ESCC) represent malignant tumors originating from the squamous epithelium lining the esophagus and account for more than 90% of esophageal malignancies in Asian populations (1). In recent years, the combination of neoadjuvant therapy followed by radical surgery has emerging as the gold-standard treatment paradigm for locally advanced ESCC, with clinically significant improvements in survival outcomes now being consistently observed. The emergence of immune checkpoint inhibitors (ICIs), specifically monoclonal antibodies targeting the PD-1/PD-L1 immune regulatory axis, has revolutionized therapeutic paradigms in advanced ESCC (2). Currently, multiple clinical studies are actively exploring the application of immunotherapy in neoadjuvant therapy. Neoadjuvant immunochemotherapy (nICT) not only demonstrates the ability to enhance pathologic complete response (pCR) rates (3, 4) but also delivers a more favorable long-term prognosis relative to neoadjuvant chemoradiotherapy (nCRT) (5).

The value of predicting pCR prior to neoadjuvant immunotherapy lies not in denying surgery to potential non-responders, but in enabling more precise risk stratification and supporting personalized adjuvant therapy decision-making in advance. Multiple studies confirm that pCR is associated with improved overall survival (OS) and recurrence-free survival (RFS). Blum MM et al. reported that in the MD Anderson cohort, patients with pCR had significantly longer median OS (71.3 vs. 35.9 months) and RFS (70.8 vs. 26.1 months) compared to non-pCR patients (6). Wu et al. reported a 5-year OS of 84.5% in pCR patients vs. 52.9% in non-pCR patients after neoadjuvant chemotherapy (7). Moreover, pCR status can assist in identifying patients who may benefit from treatment de-escalation or intensified adjuvant therapy (7). Non-pCR patients, especially those with poor response, may benefit from additional systemic therapy or closer surveillance. For patients considered unsuitable for surgical intervention, achieving pCR may represent a primary therapeutic objective (8). In such scenarios, chemo-immunotherapy could serve as a potential treatment option. Therefore, it is crucial to accurately predict the pCR to nICT and identify priority populations for nICT to avoid unnecessary adverse events and costs.

Currently, the predictive biomarkers capable of stratifying pCR and assessing survival outcomes for nICT in ESCC remain to be established. Although some biomarkers seem valuable, such as CD8+ T cell infiltration, programmed cell death ligand-1 (PD-L1) expression, and tumor mutational burden (TMB) (9), their clinical significance remains limited. Endoscopic ultrasound (EUS) serves as a critical imaging modality in the staging of ESCC (10). Emerging evidence demonstrates that maximal esophageal wall thickness and tumor volume regression rate derived from EUS, could serve as independent prognostic indicators for ESCC following neoadjuvant therapy (11). Machine learning (ML), a core subfield of artificial intelligence (AI), enables algorithms to autonomously learn from complex datasets, discern intricate biological patterns, and derive data-driven insights (12, 13). This study aimed to develop and validate a novel interpretable multimodal ML model integrating EUS features and laboratory biomarkers to pre-therapeutically predict histological response in ESCC patients receiving nICT. By incorporating the SHapley Additive Explanation (SHAP) method, we quantified feature importance and interpreted the model’s predictions to elucidate the clinical implications of the model’s ability to forecast histological outcomes following nICT and providing valuable insights for personalized therapeutic decision-making in ESCC.

## Materials and methods

### Study cohort

This retrospective study included 140 consecutive patients with ESCC at Sun Yat-sen University Cancer Center (SYSUCC, Guangzhou, China) between July 1, 2021, and July 1, 2024, who received neoadjuvant immunochemotherapy. The inclusion criteria were defined as follows: (1) histologically confirmed ESCC with clinical staging classified as cT3–4aNanyM0 or cT1–2N+M0; (2)completion of at 1–2 cycles of neoadjuvant chemoimmunotherapy;(3)subsequent esophagectomy following the completion of neoadjuvant chemoimmunotherapy. Exclusion criteria comprised: (1) Prior or synchronous malignant tumors (n=3); (2) Patients who received neoadjuvant immunotherapy at external hospitals and lacked baseline laboratory test data (n=23). After screening, a total of 114 eligible ESCC patients were ultimately included, which was randomly divided into a training set (81 patients) and a test set (33 patients) in a 7:3 ratio. This study was approved by the Ethics Committee at Sun-Yat sen University Cancer Center (Guangzhou, China; Approval No: SL-B2025-111-01).The requirement for informed consent was waived by the institutional review board given the retrospective design and complete anonymization of all patient data.

### Procedures

**Figure 1:**
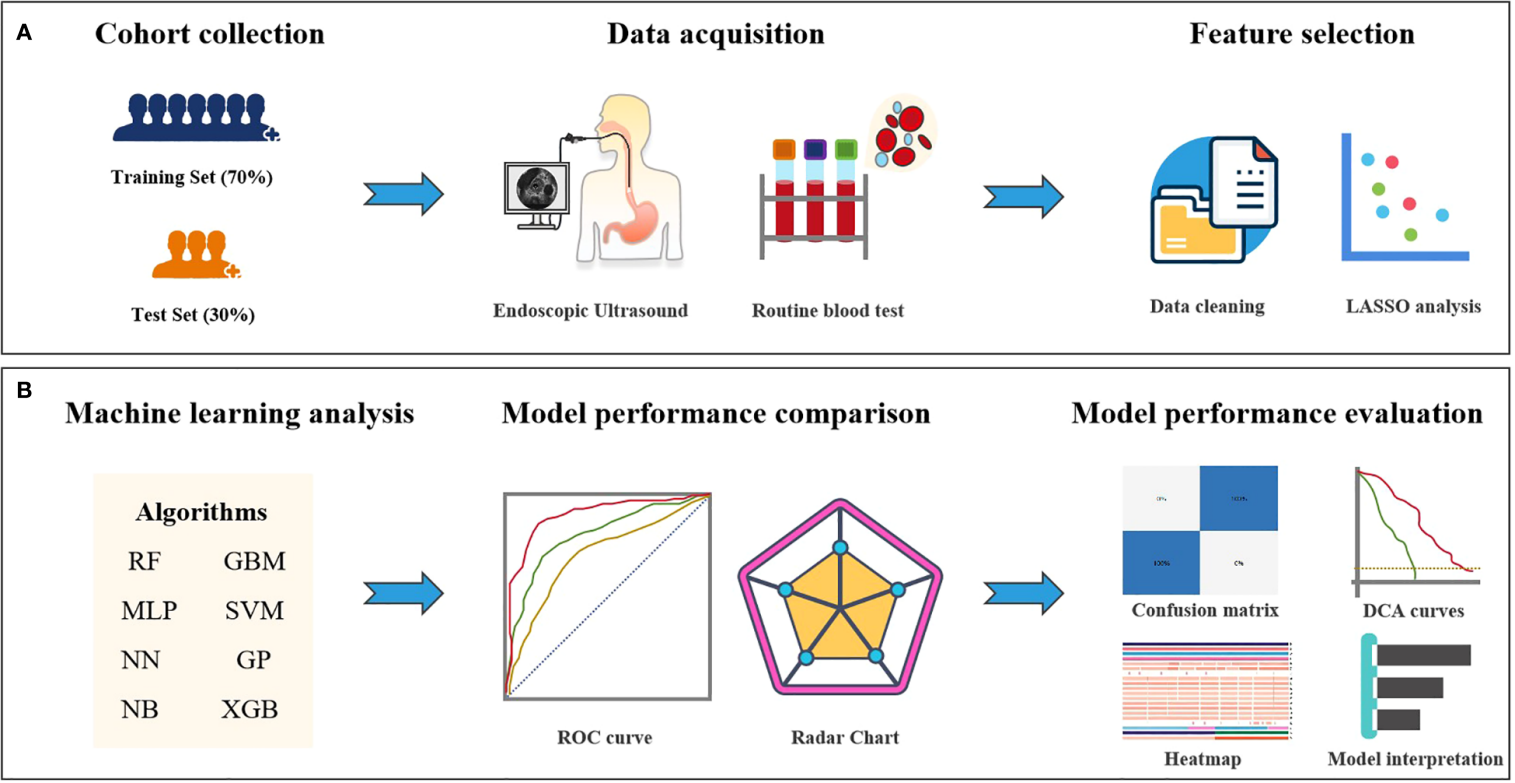
Study design flowchart. **(A)** Feature selection workflow for the machine learning model. **(B)** Deep learning feature extraction, prediction model training and validation, and quantitative analysis and evaluation.

The study design schematic is presented in **Figure 1**. We retrospectively collected clinical variables, standardized measurements from routine laboratory blood tests and EUS features that were performed on the date of or within 14 days before the first nICT treatment. All eligible patients received 1–2 cycles of ICIs (administered every 3 weeks), including pembrolizumab, nivolumab, camrelizumab, sintilimab, toripalimab, or tislelizumab, combined with chemotherapy as 1–2 cycles of platinum based doublet chemotherapy, consisting of a platinum agent (cisplatin, carboplatin, or nedaplatin) combined with paclitaxel or fuorouracil. Features with a missing percentage <10% were retained. Among all of the retained variables, the overall rate of missing data was 4.97%, with missing values imputed using the missForest algorithm. Our structured database ultimately included 65 clinical variables as candidate predictors. Pathological response was assessed and confirmed by consensus of two blinded pathologists. This study applied the pathological evaluation criteria for esophageal cancer after neoadjuvant therapy, as recommended by the College of American Pathologists (CAP) and the National Comprehensive Cancer Network (NCCN), to grade histological responses. The criteria were defined as follows: Grade 0 (complete response): No viable cancer cells in primary lesions or lymph nodes, Grade 1 (moderate response): Residual single or small clusters of cancer cells, Grade 2 (partial response): Residual cancer foci with stromal fibrosis, Grade 3 (poor response): Minimal or no tumor cell regression. Based on this classification, Grade 0 was defined as pathological complete response (pCR), while Grades 1–3 were classified as pathological incomplete response (non-pCR). This framework enabled systematic evaluation of neoadjuvant immunotherapy efficacy differences.

### Model development and validation

We performed feature selection using the least absolute shrinkage and selection operator (LASSO) regression thereby enhancing prediction accuracy and increasing model stability through elimination of non-predictive features. Eight ML models, including Random Forest (RF), Gradient Boosting Machine (GBM), Multilayer Perceptron (MLP), Support Vector Machine (SVM), Neural Network (NN), Gaussian Process (GP), Naive Bayes (NB), and Extreme Gradient Boosting (XGB) were used to predict the pathological response of nICT in ESCC patients. The final hyperparameters for each prediction model were optimized using the optimal feature subset, employing 5 repetitions of 5-fold cross-validation coupled with the default hyperparameter grid search provided by the “caret” package.

### Model performance comparison

The predictive performance of the models was evaluated using established metrics, including the area under the receiver operating characteristic curve (AUC), sensitivity, specificity, positive predictive value (PPV), negative predictive value (NPV), and accuracy. The optimal predictive model was selected based on the highest AUC in both training and test sets. Calibration of the optimal model was subsequently validated using calibration curves and quantitatively via the Hosmer-Lemeshow test to evaluate the agreement between predicted probabilities and observed outcomes, whereas decision curve analysis (DCA) was employed to quantify clinical utility by estimating net benefit across a range of threshold probabilities.

### Model explanation

Interpreting ML models remains a complex task. To address the “black box” dilemma, the SHAP framework employs game-theoretic principles to quantify feature significance and elucidate predictive outputs. This approach enables both instance-specific and overall model interpretation by quantifying individual feature contributions to predictions, thereby enhancing transparency and explainability of algorithmic decision-making processes.

### Statistical analysis

Statistical analyses were conducted using R (version 4.3.1) and SPSS software (version 18.0). Normally distributed continuous variables are expressed as mean ± SD and compared using Student’s t-test. Non-normally distributed continuous data are reported as median (IQR) with Mann-Whitney U tests. Categorical variables are presented as frequencies (%) and analyzed by chi-square tests. Feature selection employed LASSO regression (R “glmnet” package). Machine learning models were implemented via the “caret” package in R, which provides a unified interface for algorithmic implementation using specified methods: RF (method=“ranger”), GBM (method=“gbm”), MLP (method=“mlp”), SVM (method=“svmRadial”), NN (method=“NN”), GP (method=“gausspr Radial”), NB (method=“native_bayes”), XGB (method=“xgbTree”). Model performance was evaluated by ROC analysis (R “pROC” package). Comparative performance across metrics was visualized using radar plots. The optimal model’s classification results (TP/TN/FP/FN) were displayed in confusion matrices for training and test sets. Clinical utility was assessed via decision curve analysis (DCA) quantifying net benefit across threshold probabilities. Statistical significance was defined as two-tailed P < 0.05.

## Results

### Patient characteristics

The training set comprised 81 patients (median [IQR] age, 61 [58–66] years; 61 male [79.01%]), including 41 smokers (50.62%) and 22 alcohol consumers ((27.16%). Clinical staging distribution was: stage II (n=7, 8.64%), stage III (n=50, 61.73%), and stage IV (n=24, 29.63%). Following nICT, all patients underwent surgery, with pCR achieved in 36 cases ((44.44%) based on the pathological assessment of the surgical specimens. The test set included 33 patients (median [IQR] age, 64 [58–68] years; 30 males [90.91%]), with 19 smokers (57.58%) and 13 alcohol consumers (39.39%). Stage distribution was: II (n=6, 18.18%), III (n=18, 54.55%), and IV (n=9, 27.27%). The pCR was observed in 15 patients (45.5%).There were no significant differences in baseline clinical characteristics between the training and test sets. The demographic and clinicopathological characteristics of all patients are shown in **Table 1**.

**Table 1:** Patient characteristics in the training and test cohort.

Variable	Total (n=114)	Training cohort (n=81)	Test cohort (n=33)	*P*
Age (years), median (IQR)	62(58,67)	61(58,66)	64(58,68)	0.224
BMI(kg/m^2^) median (IQR)	21.95(20.13,23.69)	21.72(19.87,23.25)	22.41(20.79,24.98)	0.104
Sex				0.130
Male	94(82.46%)	64(79.01%)	30(90.91%)	
Female	20(17.54%)	17(20.99%)	3(9.09%)	
Smoking				0.500
Never	54(47.37%)	40(49.38%)	14(42.42%)	
Prior or current	60(52.63%)	41(50.62%)	19(57.58%)	
Alcohol				0.199
Never	79(69.30%)	59(72.84%)	20(60.61%)	
Prior or current	35(30.70%)	22(27.16%)	13(39.39%)	
ECOG				0.480
0	6(5.26%)	3(3.70%)	3(9.09%)	
1	108(94.74%)	78(96.30%)	30(90.91%)	
Tumour location				0.957
upper	4(3.51%)	3(3.70%)	1(3.03%)	
middle	74(64.91%)	53(65.43%)	21(63.64%)	
lower	36(31.58%)	25(30.86%)	11(33.33%)	
EUS T staging				0.658
1	1(0.88%)	1(1.23%)	0(0.00%)	
2	24(21.05%)	17(20.99%)	7(21.21%)	
3	81(71.05%)	56(69.14%)	25(75.76%)	
4	8(7.02%)	7(8.64%)	1(3.03%)	
EUS N staging				0.311
0	7(6.14%)	5(6.17%)	2(6.06%)	
1	31(27.19%)	18(22.22%)	13(39.39%)	
2	50(43.86%)	38(46.91%)	12(36.36%)	
3	26(22.81%)	20(24.69%)	6(18.18%)	
TNM stage				0.347
II	13(11.40%)	7(8.64%)	6(18.18%)	
III	68(59.65%)	50(61.73%)	18(54.55%)	
IV	33(28.95%)	24(29.63%)	9(27.27%)	
Differentiation				0.283
Poor	33(28.95%)	20(24.69%)	13(39.39%)	
Moderate	76(66.67%)	57(70.37%)	19(57.58%)	
Well	5(4.39%)	4(4.94%)	1(3.03%)	
Elevated lesion				0.166
Yes	95(83.33%)	65(80.25%)	30(90.91%)	
No	19(16.67%)	16(19.75%)	3(9.09%)	
Ulcerative lesion				0.492
Yes	61(53.51%)	45(55.56%)	16(48.48%)	
No	53(46.49%)	36(44.44%)	17(51.52%)	
Circumferential involvement				0.601
Yes	80(70.18%)	58(71.60%)	22(66.67%)	
No	34(29.82%)	23(28.40%)	11(33.33%)	
Tumor length(mm) median (IQR)	5.00(5.00,7.00)	5.00(4.00,7.00)	5.00(5.00,7.00)	0.965
Tumor thickness(mm) median (IQR)	12.35(9.63,15.90)	12.00(8.97,15.85)	13.2(10.15,16.05)	0.233
pCR				0.922
Present	51(44.74%)	36(44.44%)	15(45.45%)	
Absent	63(55.26%)	45(55.56%)	18(54.55%)	

### Predictor variable selection

**Figure 2:**
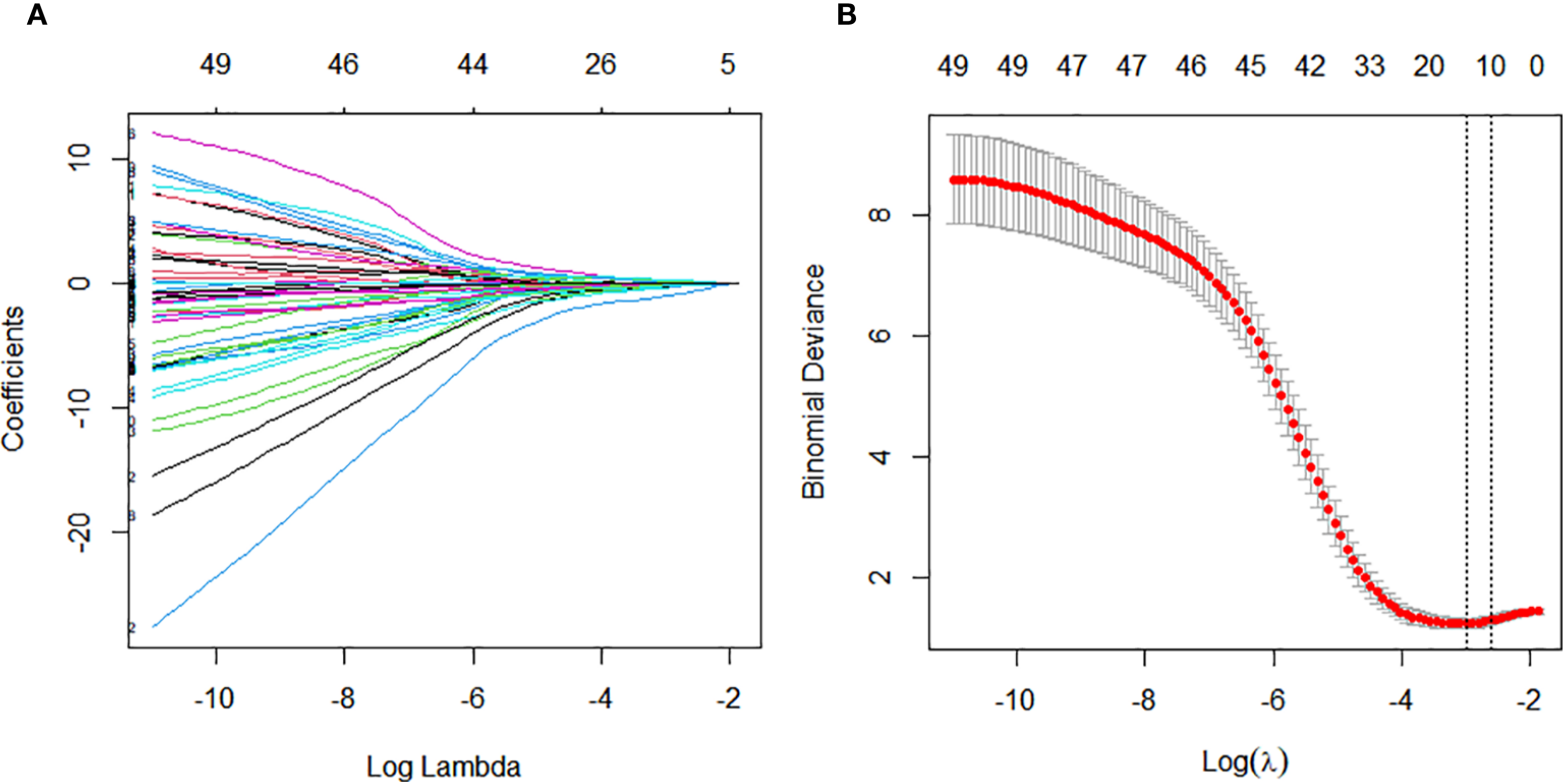
Variable selection for constructing the pathological complete response (pCR) prediction model was performed using Least Absolute Shrinkage and Selection Operator (LASSO) regression and stepwise regression. **(A)** Tuning parameter (λ) selection in the LASSO regression using 10-time cross-validation. **(B)** Coefficient profiles from the LASSO regression of the extracted features.

We performed feature selection in the training cohort to identify predictive factors associated with pathological response to nICT in ESCC. LASSO regression analyzed 65 candidate features, including 9 clinical indicators, 49 laboratory blood test parameters, and 7 endoscopic ultrasonography characteristics. This identified 17 significant predictors: alcohol consumption, circumferential involvement(CI), neutrophil-to-lymphocyte ratio (NLR), C-reactive protein (CRP), alanine aminotransferase (ALT), uric acid (UA), free thyroxine (FT4), cholesterol (CHE), creatine kinase(Ck), thyroglobulin antibody (ATPO), differentiation, albumin/globulin ratio (AGR), male, total bile acids (TBA), alkaline phosphatase (ALP), low density lipoprotein (LDL), glutamylamino transferase (GGT). (**Figures 2A, B**).

### Model development and predictive performance

**Figure 3:**
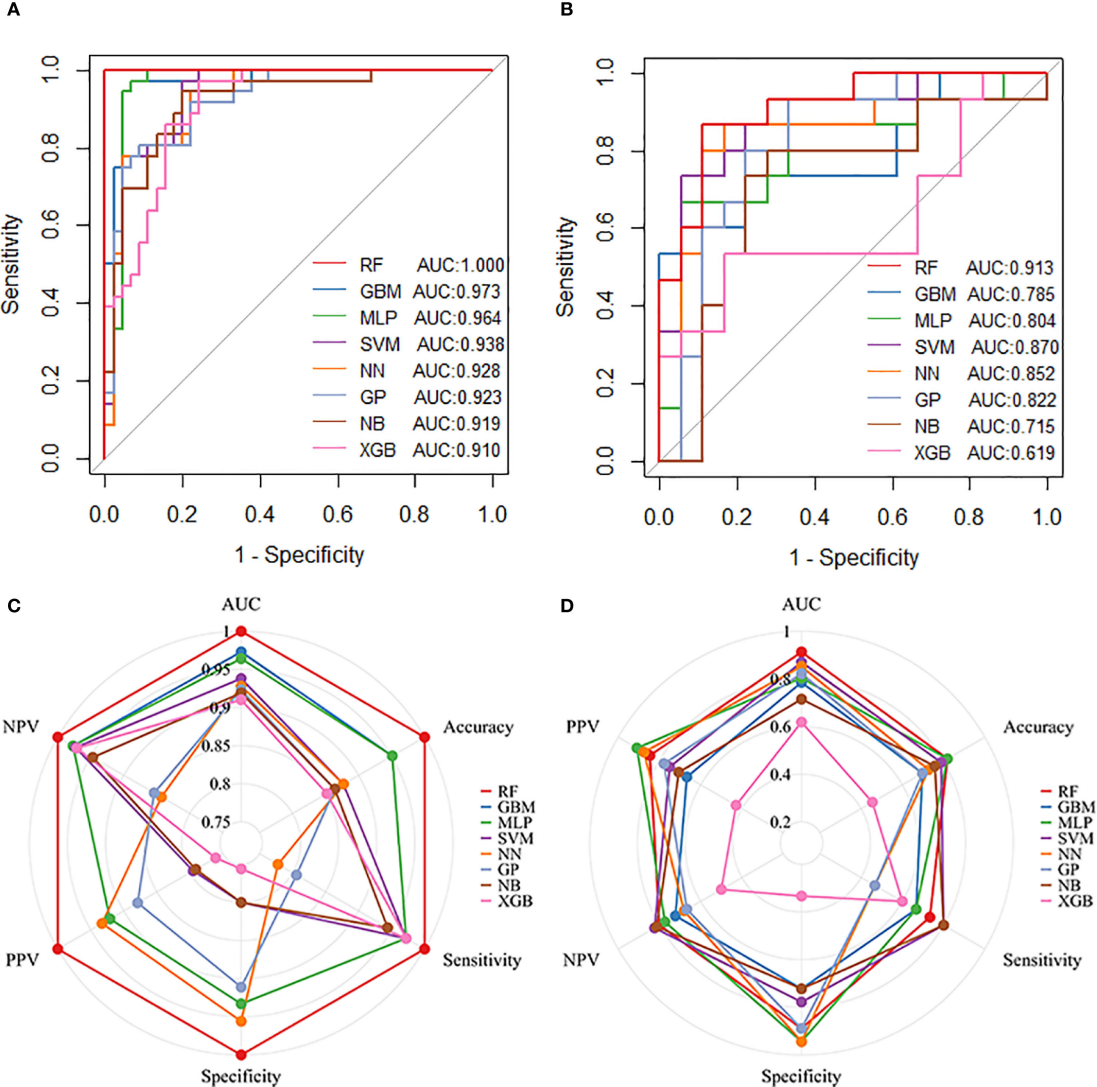
Performance of machine learning models in predicting pathological complete response (pCR) following nICT in ESCC patients. **(A)** ROC curves of eight machine learning models in the training cohort. **(B)** ROC curves of the eight models in the test cohort. **(C)** Radar plot comparing model performance metrics in the training cohort. **(D)** Radar plot comparing model performance metrics in the test cohort. Abbreviations: RF, Random Forest; GBM, Gradient Boosting Machine; MLP, Multilayer Perceptron; SVM, Support Vector Machine; NN, Neural Network; GP, Gaussian Process; NB, Naive Bayes; XGB, Extreme Gradient Boosting.

Eight machine learning models were developed using ten iterations of 10-fold cross-validation. The RF model demonstrated optimal performance in the training cohort (AUC = 1.000, sensitivity = 1.000, specificity = 1.000, PPV = 1.000, NPV = 1.000), followed by GBM (AUC = 0.973, sensitivity = 0.972, specificity = 0.933, PPV = 0.921, NPV = 0.977) and MLP (AUC = 0.964, sensitivity = 0.972, specificity = 0.933, PPV = 0.921, NPV = 0.977) (**Figures 3A, C**). RF maintained robust discrimination in the independent validation cohort (AUC = 0.913, sensitivity = 0.733, specificity = 0.889, PPV = 0.846, NPV = 0.800) (**Figures 3B, D**). **Table 2** presents the performance parameters of the eight machine learning models in the training and validation sets. These results establish RF as the optimal computational framework for predicting pCR following nICT in ESCC patients.

**Table 2:** Performance parameters of the eight machine learning prediction models in the training and test set.

Model	AUC	Accuracy	Sensitivity	Specificity	PPV	NPV
Training set						
RF	1.000	1.000	1.000	1.000	1.000	1.000
GBM	0.973	0.951	0.972	0.933	0.921	0.977
MLP	0.964	0.951	0.972	0.933	0.921	0.977
SVM	0.938	0.877	0.972	0.800	0.795	0.973
NN	0.928	0.877	0.778	0.956	0.933	0.843
GP	0.923	0.864	0.806	0.911	0.879	0.854
NB	0.919	0.864	0.944	0.800	0.791	0.947
XGB	0.910	0.852	0.972	0.756	0.761	0.971
Test set						
RF	0.913	0.818	0.733	0.889	0.846	0.800
GBM	0.785	0.697	0.667	0.722	0.667	0.722
MLP	0.804	0.818	0.667	0.944	0.909	0.773
SVM	0.870	0.788	0.800	0.778	0.750	0.824
NN	0.852	0.727	0.467	0.944	0.875	0.680
GP	0.822	0.697	0.467	0.889	0.778	0.667
NB	0.715	0.758	0.800	0.722	0.706	0.813
XGB	0.619	0.455	0.600	0.333	0.429	0.500

**Figure 4:**
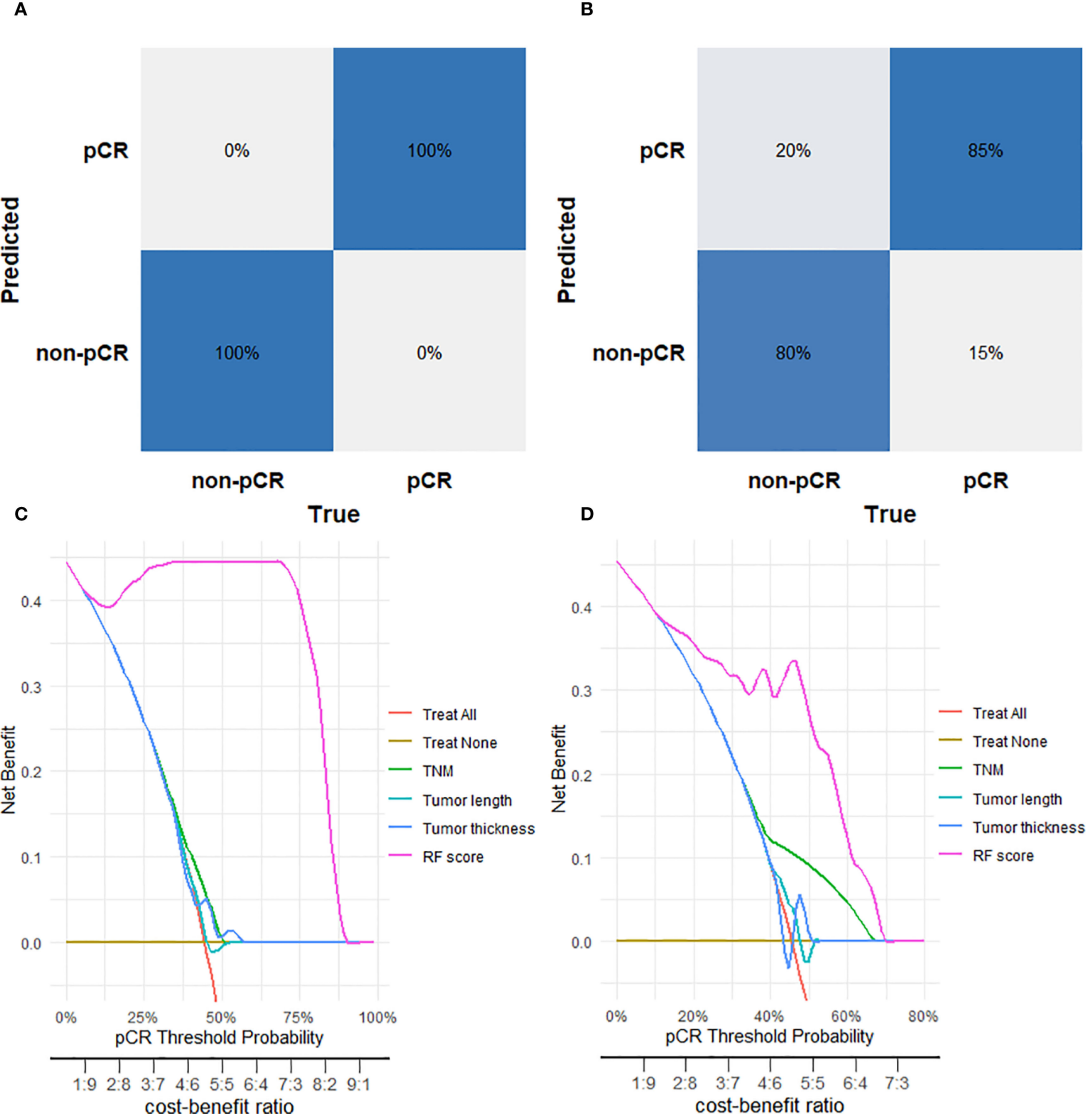
Performance evaluation of the Random Forest (RF) model for predicting pathological complete response (pCR). **(A)** Confusion matrix for the RF model in the training cohort. **(B)** Confusion matrix for the RF model in the tes cohort. **(C)** Decision curve analysis for the RF model in the training cohort. **(D)** Decision curve analysis for the RF model in the test cohort.

Confusion matrix analysis showed the RF model achieved 100% true prediction rate in the training set (**Figure 4A**) and 85% in the validation set (**Figure 4B**). Calibration curve analysis was employed to evaluate the model’s predictive reliability, quantifying the concordance between predicted probabilities and observed outcomes. The RF model showed excellent calibration fidelity in both training and test cohorts. (**Supplementary Figure S1**). For the training set the Brier score was 0.0304, and the Hosmer-Lemeshow test yielded a chi-square value of 13.44 (p=0.0976). For the validation set, the Brier score was 0.1623, while the Hosmer-Lemeshow test showed a chi-square value of 8.19 (p=0.0848). Both datasets demonstrate good agreement between predicted probabilities and observed outcomes. Decision curve analysis further indicated that the RF model provided greater net clinical benefit across the entire threshold probability range (0–80%) compared with TNM stage, tumor length, and tumor thickness in both training (**Figure 4C**) and test (**Figure 4D**) sets.

### Heatmap analysis of RF model variables

**Figure 5:**
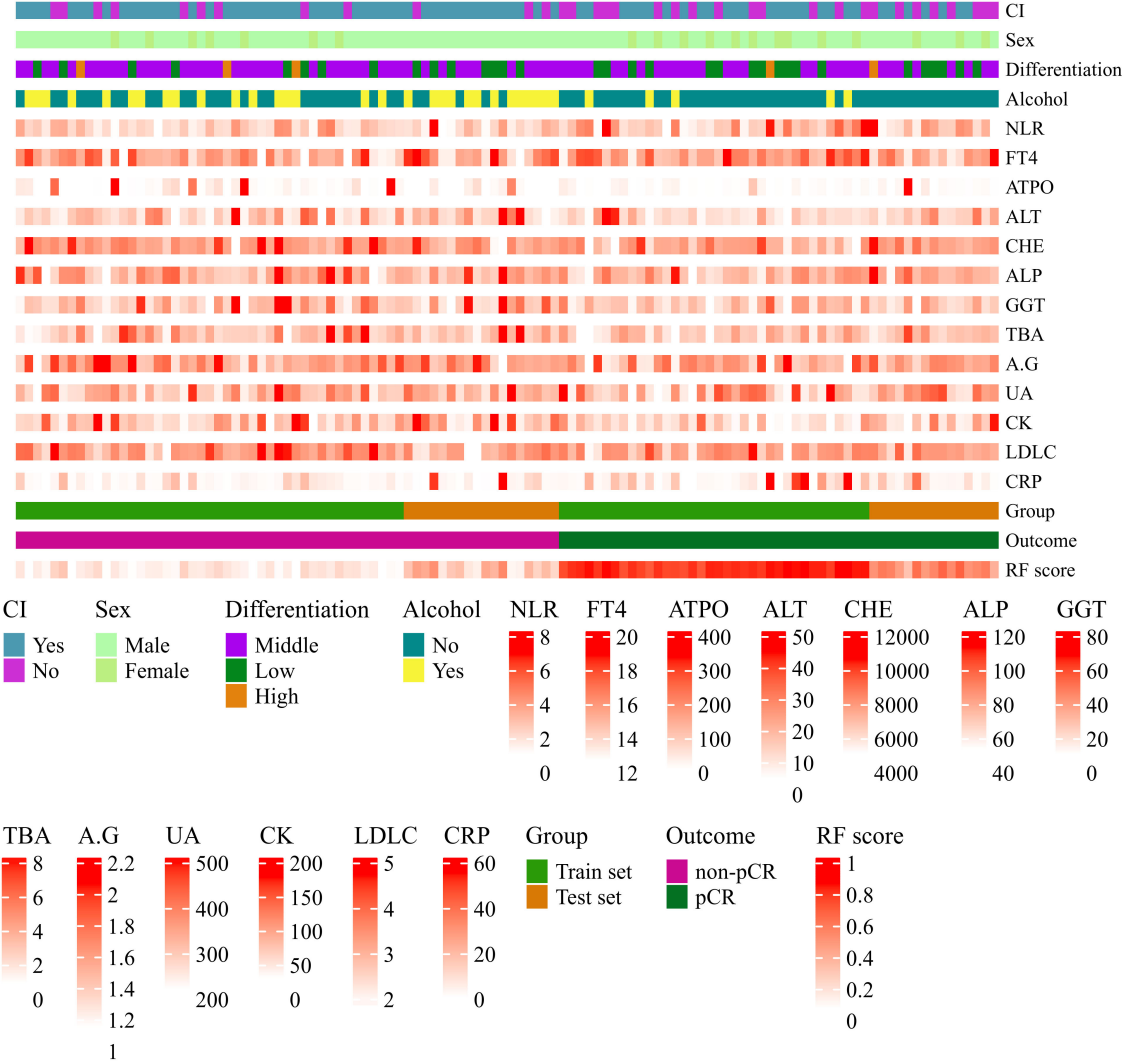
Heatmap visualization of variables associated with pathological complete response (pCR) prediction in ESCC patients after nICT using the Random Forest (RF) model. Each row represents a variable; each column represents a patient sample. Continuous variables are represented by a color gradient and categorical variables use distinct colors per category. Variables include circumferential involvement, gender, pathological differentiation degree, alcohol consumption, neutrophil-to-lymphocyte ratio (NLR), free thyroxine (FT4, pmol/L), anti-thyroid peroxidase antibody (A-TPO, U/ml), alanine aminotransferase (ALT, U/L), cholinesterase (CHE, U/L), alkaline phosphatase (ALP, U/L), gamma-glutamyl transferase (GGT, U/L), total bile acid (TBA, umol/L), albumin-to-globulin ratio (A/G), uric acid (UA, umol/L), creatine kinase (CK, U/L), low-density lipoprotein cholesterol (LDL-C, mmol/L), C-reactive protein (CRP, mg/L), RF model predicted probability (RF score), actual pathological response outcome (Outcome), and dataset grouping (Group).

A heatmap was constructed to visually characterize the discriminatory capacity of the RF model in predicting pCR following nICT in ESCC patients. The multivariate visualization matrix employed color gradients to depict the spatial distribution of the predictive variables across the ESCC cohort, while simultaneously mapping algorithm-derived pCR probability scores against histologically confirmed treatment outcomes. Differential clustering patterns emerged between pCR and non-pCR cohorts (**Figure 5**), with the RF model maintaining high predictive fidelity in both training and test cohorts. This demonstrates robust generalizability of computationally derived prognostic signatures, suggesting clinical utility for early identification of nICT responders.

### Model explanation

**Figure 6:**
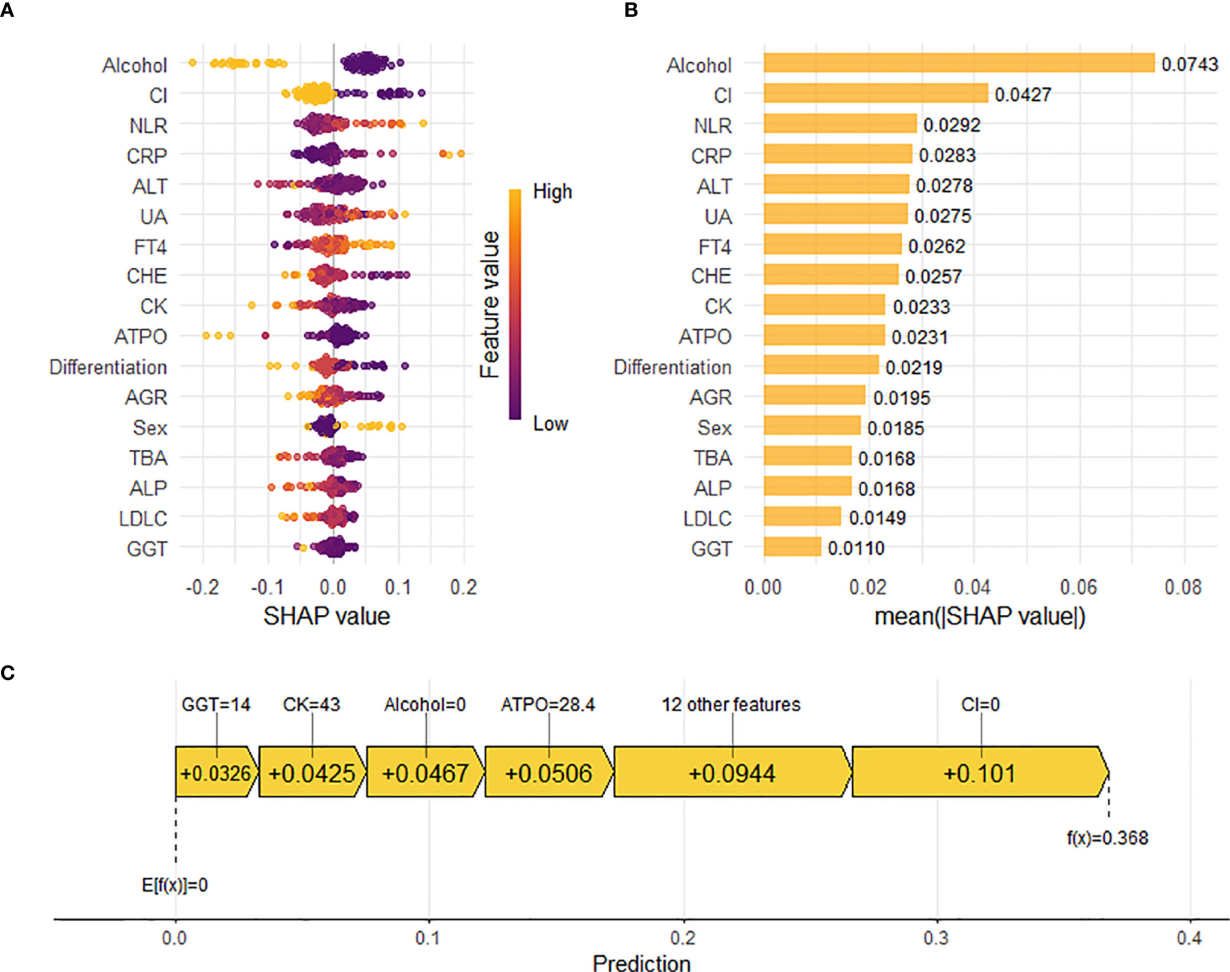
Interpretation of the Random Forest (RF) model for predicting pathological complete response (pCR) using SHapley Additive exPlanations (SHAP) analysis. **(A)** SHAP summary dot plot. Features are ranked by descending mean absolute SHAP value, representing their overall importance. Each point represents the SHAP value for a feature in an individual patient. Color indicates the relative value of the feature (orange: high, purple: low). Vertical dispersion reflects data density. **(B)** SHAP summary bar plot. Features are ranked by descending mean absolute SHAP value, representing their average magnitude of contribution to the model’s predictions. **(C)** SHAP waterfall plot. Illustrates the cumulative contribution of individual features to shifting the model’s expected output (base value, E[f(X)]) to the final prediction (f(x)) for a representative patient (e.g., Patient 3). Feature values and their corresponding SHAP values are annotated. Positive SHAP values indicate features pushing the prediction towards pCR.

To elucidate the underlying decision-making process of the RF model, we employed the SHAP method for model interpretability. SHAP analysis quantifies the marginal contribution of each feature to the prediction by computing Shapley values, enabling a comprehensive assessment of feature-specific impacts on the model’s output. The SHAP summary dot plot (**Figure 6A**) visually shows the direction and strength of the influence of each feature on the global interpretability of the RF model. In addition, the SHAP bar plot (**Figure 6B**) facilitates intuitive comparison of feature importance by displaying mean absolute SHAP values. Key predictors included alcohol, circumferential involvement, high NLR,high CRP and high ALT exhibited the highest Shapley values, underscoring their pivotal roles in the model’s predicting pCR following nICT in ESCC patients.

Beyond global feature importance, we utilized the SHAP plot illustrates the local contributions of individual features to the RF model predictions. **Figure 6C** reveals the specific impact of each feature on the predicted probability of pCR for individual patients. For example, in a patient with GGT level of 14 U/L, CK level of 43 U/L, no history of alcohol consumption, A-TPO level of 28.4 U/ml, and absence of circumferential involvement, the corresponding Shapley values of +0.0326, +0.0425, +0.0467, +0.0506, and +0.101, respectively, indicated positive contributions to the pCR prediction. Meanwhile, other features, including gender, pathological differentiation, ALT level, and uric acid (UA) level, also exerted varying degrees of influence on the model’s decision-making process.The waterfall plot provided a holistic view of how different features interacted and contributed to the final prediction, thereby offering valuable insights into the complex relationships between clinical variables and treatment outcomes in ESCC patients receiving nICT.

## Discussion

This study presents a deep learning-derived model for the early prediction of pCR in ESCC patients receiving neoadjuvant immune checkpoint therapy. By integrating clinical indicators, laboratory biomarkers, and endoscopic ultrasonography features, we identified predictive biomarkers and systematically evaluated eight machine learning models for pCR prediction. The random forest algorithm demonstrated superior predictive accuracy across both in the training cohort (AUC 1.000) while maintaining robust performance in the test cohort (AUC 0.913), outperforming traditional clinical indices. Notably, we derived a novel probabilistic scoring system from the RF model that revealed significant differences between pCR and non-pCR groups across all patient strata. This clinically applicable tool provides accurate pCR prediction prior to treatment completion, potentially identifying candidates most likely to benefit from nICT. Such stratification may mitigate overtreatment risks while advancing personalized therapeutic strategies for ESCC.

In recent years, immunotherapy has emerged as a revolutionary oncologic therapy, demonstrating particularly pronounced advantages in neoadjuvant settings for ESCC. Neoadjuvant immunotherapy has transformed ESCC management through immune checkpoint inhibitors (ICIs) administered preoperatively to induce tumor regression, downstage clinical disease, and improve complete resection rates (14). Clinical evidence shows that adding immunotherapy to doublet chemotherapy or chemoradiotherapy further improves treatment outcomes. The ESCORT-NEO/NCCES01 trial notably demonstrated a significant increase in pathologic complete response (pCR) rates with immunochemotherapy, achieving 28.0% and 15.4% pCR rates in combination groups versus 4.7% in the chemotherapy-alone arm (15). Furthermore, Yu et al. reported that the nICT group had a better 3-year disease-free survival rate (87.4% vs 72.8%) and 3-year OS rate (91.7% vs 79.8%) compared with the nCRT group (5). Critically, Patients who achieve pCR may benefit from organ-preserving strategies, avoiding radical esophagectomy. Accurate assessment of residual disease after neoadjuvant therapy is critical for implementing such strategies. However, current non-invasive methods are unable to reliably identify pCR, creating a critical clinical unmet need for pretreatment predictive tools.

Tumor mutational burden (TMB), microsatellite instability (MSI), and PD-L1 expression have been investigated as potential biomarkers for immunotherapy response, yet their clinical utility remains controversial (16). While two independent clinical trials reported significant associations between pretreatment TMB levels and response to neoadjuvant immunotherapy (17, 18), another study found no correlation between TMB and nICT efficacy (19). Additionally, PD-L1 expression faces similar challenges as a predictive marker. The trials such as KEYNOTE-590 (2), CheckMate-648 (20), and JUPITER-06 (21) have demonstrated that ESCC patients patients derive clinical benefit from immunotherapy irrespective of PD-L1 status, yet the TD-NICE study failed to establish a significant correlation between PD-L1 expression and pCR (4). The inconsistent predictive performance of current biomarkers arises from tumor immune microenvironment complexity, necessitating composite biomarker integration for accurate nICT outcome prediction in patients with ESCC.

Our study integrated pre-neoadjuvant immunochemotherapy features from ESCC patients, including endoscopic ultrasonography delineating local tumor characteristics, peripheral blood biomarkers reflecting systemic immune status, and clinical parameters capturing baseline host factors. Subsequent multimodal fusion of local-systemic-host data overcomes the spatiotemporal limitations of conventional response assessment methods, with the RF algorithm demonstrating exceptional accuracy in predicting pCR for ESCC patients receiving nICT. SHAP analysis quantified feature contributions to pCR prediction, identifying alcohol, circumferential involvement, high NLR,high CRP and high ALT as the top five predictors. The directional influence and quantitative impact of individual features exhibited dynamic shifts upon their intrinsic values and combinatorial interactions with co-occurring variables, highlighting the demonstrating the model’s complexity in predicting ICI efficacy for each patient. Then, we explored the relationship of these features and ICI efficacy. Firstly, the development of ESCC demonstrates a strong correlation with ethanol intake (22). Epidemiological evidence indicates that regular consumption of alcoholic beverages elevates ESCC risk by approximately 60% (23). Furthermore, the relationship between chronic alcohol exposure and ESCC risk has been shown to exhibit a dose-response relationship. Sustained excessive alcohol consumption is associated with substantially elevated risks of both disease incidence and mortality rates (24). Mechanistically, experimental studies suggest that ethanol metabolites may impair T lymphocyte activation pathways, thereby compromising antitumor immunity through immunosuppressive mechanisms (25). Moreover, Fu et al. (26) highlight that Aldehyde dehydrogenase 2 (ALDH2) is a key enzyme involved in alcohol metabolism, alcohol consumption could induce ALDH2 and subsequently upregulate PD-L1 expression in CRC to allow their escape from immune surveillance. In summary, alcohol consumption may compromise patient responsiveness to nICT by modulating T cell differentiation or regulating PD-L1 expression. Secondly, circumferential involvement ≥(1/2) of the circumference is a risk factor for postoperative stenosis in endoscopic submucosal dissection of ESCC. One possible reason is that circumferential involvement might promoting fibrosis and scar formation in the esophageal wall, ultimately leading to esophageal stricture, and significantly affecting patient prognosis (27); the other possible reason is that circumferential involvement might alter local blood supply and lymphatic structure, resulting in insufficient drug penetration depth to reach the tumor core; furthermore, tumors with a small circumferential invasion range may preserve more intact lymphatic structures and vascular networks, facilitating the infiltration of effector T cells (such as CD8+T cells). Studies have shown that patients with an immune-enriched TME (highly infiltrated lymphocytes, activated IFN-γ signaling) at baseline exhibit better responses to neoadjuvant immunotherapy, with significantly increased pCR rates; lastly, High proportion of exhausted precursor T cells (Tpex): Tumors with minimal circumferential invasion may be enriched with SPRY1+PD1+CD8+T cells (exhausted precursor cells with stem-like properties), which can be activated and expanded by PD-1/PD-L1 inhibitors, driving potent anti-tumor immune responses. However, its relationship with neoadjuvant therapy remains unclear. Thirdly, we reported that high NLR, CRP and ALT were related to the poor prognosis of nICT. In previous studies on inflammatory responses, NLR (28), CRP (29), and ALT (30), as reliable and easily accessible indicators of immune-inflammatory reactions, have been demonstrated to play significant predictive roles in various diseases, including multiple solid tumors such as esophageal cancer, and are commonly used to assess the severity of systemic inflammatory responses. As is well known, the formation of esophageal strictures requires the involvement of immune-inflammatory cells and inflammatory mediators. Therefore, inflammatory factors may serve as another predictive indicator for esophageal strictures (31).

Currently, individual research teams have developed models for predicting therapeutic efficacy following neoadjuvant therapy in ESCC patients. However, it is worth noting that all these predictive models primarily rely on the the Response Evaluation Criteria in Solid Tumors (RECIST 1.1). Since the response patterns of tumors treated with immune checkpoint inhibitors (ICIs) may differ from those of conventional therapies, pseudoprogression and mixed responses can lead to RECIST 1.1 misclassifying such cases as progressive disease (PD) during immunotherapy evaluation. Ultimately, the gold standard for efficacy assessment remains postoperative pathology. Machine learning offers a transformative solution by decoding complex biological patterns through iterative algorithmic learning from multimodal datasets. Unlike rule-based methods, ML frameworks excel at capturing nonlinear relationships and subtle feature interactions—capabilities critical for modeling the heterogeneity of the tumor immune microenvironment. In this study, we innovatively developed a RF model derived scoring system provides clinicians with an objective tool to stratify patients most likely to benefit from nICT. Furthermore, all the predictive factors included in the RF model are routine examination items for ESCC patients during hospitalization and are easily accessible, providing feasibility for the clinical application.

Although the developmen of RF model demonstrated robust predictive performance in this study, there are still some limitations. Firstly, the retrospective design may introduce selection bias despite strict inclusion and exclusion criteria, which may limit the generalizability of the prediction model. Secondly, the model was developed using data exclusively from a single Chinese medical center. Although internal validation has confirmed the predictive efficacy of the model, the relatively small sample size and lack of external validation in this study may affect the robustness and broad applicability of the prediction model. Moreover, while Random Forest achieved the highest mean AUC, its apparent advantages over most comparators were not statistically robust to multiple testing correction. This suggests these differences may represent random variations amplified by repeated comparisons. Therefore, this work as a retrospective exploratory analysis, subsequent studies should organize multicenter, prospective large-scale studies involving ESCC patients from various regions and medical institutions aimed at dynamically evaluating the predictive performance of the model in real clinical settings. Additionally, integrating multi-omics data, including genomic, radiomic, and proteomic features, holds promise for improving prediction accuracy by capturing the complex biological mechanisms underlying tumor-immune interactions, ultimately facilitating the development of a more refined and clinically useful immunotherapy prediction model.

## Conclusion

Our study established an interpretable random forest model using baseline endoscopic ultrasonography and hematological parameters that accurately predicts histological response to neoadjuvant immune checkpoint therapy in ESCC patients. Validated across independent cohorts, the model offers a clinically actionable tool for pretreatment identification of responders, thereby optimizing personalized therapeutic strategies while reducing unnecessary healthcare expenditures and mitigating immune-related adverse events through early intervention.

## Data Availability

The raw data supporting the conclusions of this article will be made available by the authors, without undue reservation. The authenticity of this article has been validated by uploading the key raw data onto the Research Data Deposit public platform (www.researchdata.org.cn), with the approval RDD number as RDDA2025408025.

## Supplementary material

Supplementary Figure 1 Calibration curves of the Random Forest (RF) model for predicting pathological complete response (pCR) in the training and test cohorts.
