# Supplementary figures and images for: An interpretable machine learning model using multimodal pretreatment features predicts pathological complete response to neoadjuvant immunochemotherapy in esophageal squamous cell carcinoma
Source: Front Immunol. 2025 Sep 16;16:1660897. doi: 10.3389/fimmu.2025.1660897 (PMC12479532; doi:10.3389/fimmu.2025.1660897)

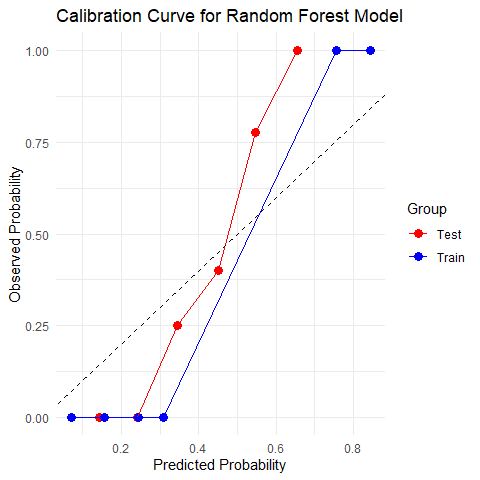

Supplement: Supplementary Figure 1 — Calibration curves of the Random Forest (RF) model for predicting pathological complete response (pCR) in the training and test cohorts. [file Image1.tiff]
